# MASI enables fast model-free standardization and integration of single-cell transcriptomics data

**DOI:** 10.1038/s42003-023-04820-3

**Published:** 2023-04-28

**Authors:** Yang Xu, Rafael Kramann, Rachel Patton McCord, Sikander Hayat

**Affiliations:** 1 UT-ORNL Graduate School of Genome Science and Technology, University of Tennessee, Knoxville, TN 37996 USA; 2 Institute of Experimental Medicine and Systems Biology, RWTH Aachen University, Aachen, Germany; 3 Department of Biochemistry and Cellular and Molecular Biology, University of Tennessee, Knoxville, TN 37996 USA; 4 Present Address: Data Sciences Platform, Broad Institute of MIT and Harvard, Cambridge, MA 02142 USA

**Keywords:** Data integration, RNA sequencing

## Abstract

Single-cell transcriptomics datasets from the same anatomical sites generated by different research labs are becoming increasingly common. However, fast and computationally inexpensive tools for standardization of cell-type annotation and data integration are still needed in order to increase research inclusivity. To standardize cell-type annotation and integrate single-cell transcriptomics datasets, we have built a fast model-free integration method, named MASI (Marker-Assisted Standardization and Integration). We benchmark MASI with other well-established methods and demonstrate that MASI outperforms other methods, in terms of integration, annotation, and speed. To harness knowledge from single-cell atlases, we demonstrate three case studies that cover integration across biological conditions, surveyed participants, and research groups, respectively. Finally, we show MASI can annotate approximately one million cells on a personal laptop, making large-scale single-cell data integration more accessible. We envision that MASI can serve as a cheap computational alternative for the single-cell research community.

## Abstract (web summary)

MASI is a computational pipeline that enables the integration and annotation of large single-cell transcriptomic datasets with limited computational resources.

## Introduction

Single-cell RNA-seq (scRNA-seq) technologies have rapidly evolved over the last decade^1–3^. Numerous studies have demonstrated the utility of single-cell transcriptomics datasets in improving our understanding of cellular heterogeneity and molecular mechanisms at unprecedented resolution. Over the past years, many single-cell datasets have been made available from different research groups, using multiple single-cell platforms, and covering diverse biological conditions. Global collaborations, for example, the Human Cell Atlas project, further make profiling millions of cells possible^4^. However, this trend of increasing data generation also introduces the challenge of data annotation and integration. Though many conventional machine learning methods^5–7^ and deep-learning-based approaches^8–11^ provide solutions to automatic annotation and integration of single-cell datasets, these methods usually require large probabilistic modeling or gradient backpropagation through a large neural network. Therefore, their availability to a wider research community is still limited due to the computational cost. Besides the need to reduce the computational burden, we also face another challenge of standardizing data annotation. Different research groups have their own practices for cell-type annotation. The same cellular system profiled by different research groups could have different cell-type annotations, in terms of naming style and annotation resolution. For example, the human heart atlas study defined 9 major cell types and 27 sub-types, while a similar atlas-level study by Tucker et al. defined 17 cell types for the cardiovascular system^12,13^. Without the standardization of cell-type annotation, it is hard to establish an agreement for integrative analyses. This is also a pressing issue for integrating COVID-19-related single-cell transcriptomics datasets, which have been generated by researchers across the globe to understand the SARS-CoV-2 disease mechanism^14,15^.

To address these issues in the integrative analysis of scRNA-seq data, we propose MASI, a fast model-free method for standardization and integration of scRNA-seq data. Our method relies on putative cell-type marker genes from the reference data to uniformly annotate and integrate query datasets. Cell-type markers should serve as reliable indicators that hold a constant truth to define cell types across different studies. Because of its simplicity, MASI can easily accommodate annotation and integration for millions of cells with limited computational resources. Relying on cell-type markers to integrate and annotate scRNA-seq data is a distinct approach, because most of the existing methods for data integration and annotation, including all methods used in the following benchmark^6,7,10,11,16,17^, are training large models either in a supervised or unsupervised manner. Our benchmark also shows that such a marker-based approach can compete against other well-established model-based annotation and integration methods. Finally, we demonstrated that MASI can standardize and integrate large-scale single-cell transcriptomics data in three cases, covering kidney, lung, and heart studies.

## Results

### Development of MASI

In our previous study, we found that converting the gene expression matrix to a cell-type score matrix through a scoring method^18^ given cell-type markers in PanglaoDB^19^ can be used for integrative cell-type annotation^20^. However, we did not further investigate what is the essential component of the integrative annotation, and we did not know if the power of the cell-type score matrix applies to more general cases of single-cell data integration. To answer these two remaining questions, we tested different data processing pipelines in multiple scRNA-seq datasets and examined how these pipelines deal with the issue of batch effects and integrate single-cell data from different sources. For this, we selected 6 batch-involved datasets from 6 different tissues and measured the impacts of 16 different data processing pipelines on revealing cell heterogeneity while mixing batches (Supplementary Fig. 1a). These 6 batch-involved datasets include mouse liver across two scRNA-seq platforms^21^, human pancreas data across 5 scRNA-seq platforms^22–26^, human hematopoietic data across 4 studies^27–30^, human heart atlas^12^, mouse primary cortex data across 3 scRNA-seq platforms^31^, and mouse brain data across 4 studies^32–35^. Of note, the human heart atlas data were collected from two institutes and covered single-cell, single-nuclei, and CD45+-enriched data. The #1 pipeline is the most basic data processing for scRNA-seq analysis, which does not take batch information into consideration for calculating the highly variable genes (HVG). The #2 pipeline differs from #1 in terms of identifying highly variable genes by batch and only including shared HVGs in downstream processing. For #3, #4, #5, and #6 pipelines, we introduced cell-type markers that are obtained from different sources, including CellMarker^36^, PanglaoDB^19^, ScType^37^, and specific reference data. We only included marker genes as features in the downstream analyses. For #7, #8, #9, and #10 pipelines, we further converted the gene expression matrix to a raw cell-type score matrix containing cell types in CellMarker, PanglaoDB, ScType, or in the specific reference data. Just as each cell has a value for the expression of each gene in the original gene expression matrix, each cell has a score for each cell type in the cell-type score matrix. The score for each cell type effectively represents the signature that the given cell belongs to that cell type. We call it a raw cell-type score matrix because it is simply summing up all marker genes for a given cell type. In pipelines #11, #12, #13, and #14, we added an additional transformation and thresholding into the process, before converting the gene expression matrix to a cell-type score matrix by summing up all marker genes for a given cell type. We call it PlinerScore because it was proposed by Pliner et al.^18^. #15 pipeline is a combination process of #2 and #14 pipelines. Deep-learning-based batch correction methods demonstrated considerable success in integrative analysis of scRNA-seq data, and we noticed that the frequent practice across these methods is the use of batch normalization layer and non-linear activation layer, which splits the whole dataset into multiple mini-batches, standardizes cells in each batch, and transforms the outcome with a non-linear activation function^8,10,11,38,39^. This batch normalization and non-linear activation process do not require weight training, and we included it as the last pipeline, #16. To evaluate the impact of these 16 pipelines on revealing cellular heterogeneity and mixing batches, we used two common metrics, cell-type silhouette score and batch mixing entropy score (Supplementary Fig. 1b). Cell-type silhouette score quantifies how the processing pipeline reveals cell-type structure, while the batch entropy mixing score measures how well batches are mixed. A decent data integration should end up with high values of both cell-type silhouette and batch-mixing entropy scores. Based on our benchmark here, we observed that pipelines that convert gene expression matrix to cell-type score matrix could largely resolve batch effects and reveal cell-type structure (from pipeline #7 to #14). This is true regardless of the source of the cell-type markers, whether from CellMarker, PanglaoDB, ScType, or specific reference data. However, calling HVG by batch (pipeline #2) and using cell-type markers (from pipeline #3 to #6) alone couldn’t remove batch effects in most cases. We also noticed that the pipelines with PlinerScore (from pipeline #11 to #14) had a slight improvement from the raw cell-type score pipelines (from pipeline #7 to #10). Both pipelines #15 and #16 have a higher batch entropy mixing score, but a lower cell-type silhouette score. These results also suggest that conversion from gene expression matrix to cell-type score matrix is the core component for integration analysis in our previous study. Pipelines #7 to #14 showed substantial improvement from pipelines #1 to #6, indicating a general usage of the cell-type score for single-cell data integration (Supplementary Fig. 1b). In summary, one can convert gene expression matrix to cell-type score matrix with CellMarker, PanglaoDB, ScType, or a specific reference data for batch-effect correction and data annotation. Besides these 3 marker databases (CellMarker, PanglaoDB, and ScType), there are also other resources that provide comprehensive or customized cell-type marker information. For instance, cell-type markers were identified from bulk RNA-seq databases like ImmGenData^40^ and HumanPrimaryCellAtlasData^41^. Though the ability to remove batch effects with the other resources is not tested here, we still encourage users to select more customized cell-type marker databases based on their single-cell data source and specific needs. Compared to pipelines from #1 to #7, paired *t*-tests also showed that Pipeline #14 has substantially better integration scores (*p*-value < 0.05). Across 6 benchmark datasets here, pipeline #14, along with #16, was ranked as one of the top processing pipelines based on integration score (Supplementary Data 1). Considering #14 is much simpler than #16, and our focus in this study is annotating and integrating scRNA-seq data with reference data, we selected pipeline #14 as the final data processing pipeline.

### Integrative analysis using cell-type score matrix

Most integration methods would learn a latent space, in which batch effects are resolved. However, learning this integrated latent space does not guarantee that true biological information is also preserved in the lower dimension^42^. The key component of our chosen pipeline #14 is the conversion of the gene expression matrix into a cell-type score matrix. This conversion should condense biological information from a high-dimension gene feature space into a lower-dimension cell-type feature space. Meanwhile, this conversion does not involve any learning but instead relies on prior knowledge. Thus, it also should preserve the intrinsic biological structure in a lower dimension without introducing distortion to the data. In our pipeline from #7 to #14, the prior knowledge sources are 3 comprehensive marker databases or markers from closely relevant reference data. Above, we showed that all pipelines which include conversion to a cell-type score matrix can correct batch effects. To test the idea that the conversion of gene expression matrix to cell-type score matrix also preserves intrinsic biological information, we next tested whether the cell-type features could construct lineages for multi-batch scRNA-Seq datasets. For this, we selected three datasets for integrative lineage analysis: (1) human peripheral blood mononuclear cell (PBMC) data of patients with Kawasaki disease obtained before and after IVIG (intravenous immunoglobulin) treatment^43^, (2) mouse brain lineage tracing at different time points^44^, and (3) zebrafish embryo from two studies that cover 13 major developmental stages^45,46^.

**Fig. 1:**
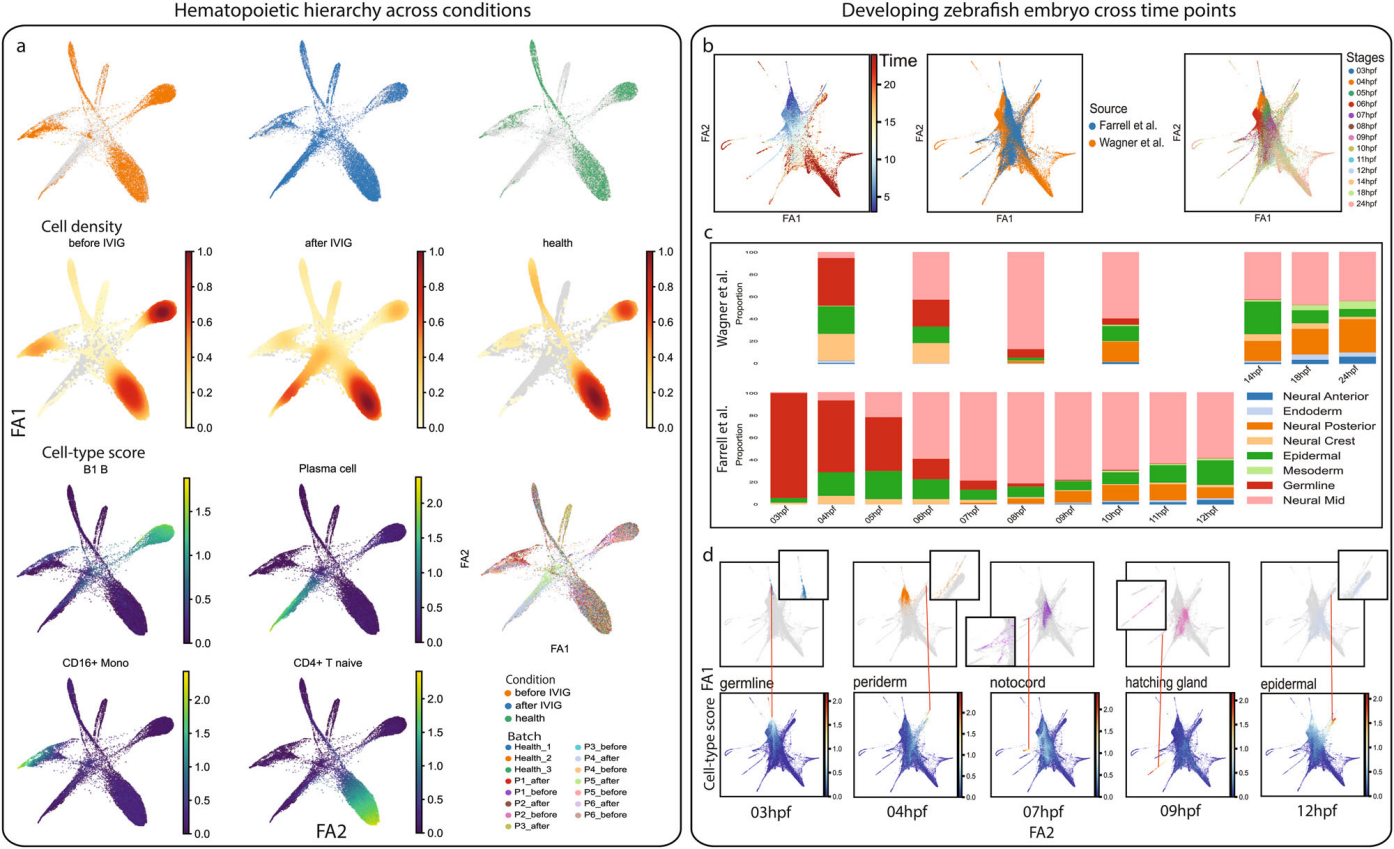
Integrative lineage analysis using cell-type score matrix. **a** Integrative lineage analysis for multi-condition human hematopoiesis study^43^. Cell density, cell-type score, and batch id for human hematopoiesis samples under different conditions are visualized separately through the first two ForceAtlas2. **b** integrative lineage analysis for two developing zebrafish embryo data^45,46^. Cells are visualized through UMAP and are colored according to developmental time (left), study id (middle), and developmental stages (right). **c** Compositions of eight major lineages along the developmental stages in zebrafish embryo. All lineages sum up to 1 in one stage, and data from the two studies are visualized separately. **d** Identification of lineage origin time. Visual investigation is conducted by matching emergence of a cell-type with the earliest developmental stage in the data.

Both human hematopoiesis and mouse brain lineage tracing studies used a multi-condition design. We were able to obtain cell-type markers from external 10X Genomics PBMC data^30^, while we used author-verified cell-type markers from the original report for the mouse brain lineage tracking study^44^. We constructed an integrative lineage map with cell-type score matrices and visualized population density and cell-type score (Fig. 1a and Supplementary Fig. 2). We can directly interpret data by visualizing cell-type scores, and we identified lineage changes in human PBMC data before and after IVIG treatment. Our identification is consistent with the original report. For example, we observed decreased B1 B-cell and CD16+ monocyte lineages as well as increased plasma cell and CD4+ T native lineages after IVIG treatment for acute Kawasaki disease patients (Fig. 1a). In mouse brain lineage tracing study, the integrative lineage map showed that mitotic progenitor cells injected at E10.5 time point tend to differentiate to astrocyte and OPC (oligodendrocyte precursor cell), while the lineage specification may shift to neuron at 14.5-time point (Supplementary Fig. 2). This is also consistent with the original findings.

Our integrative analysis of developing zebrafish embryos consists of data from two independent data sources that cover different time points of post-fertilization. Wagner et al. collected cells from 7 stages including 4, 6, 8, 10, 14, 18, and 24 hpf (hours post fertilization), while Farrell et al. designed 12 finer stages ranging from 3 to 12 hpf. We were unable to find an external marker gene reference for the two developing zebrafish datasets. Given they were in a time-series design, we reasoned that the end-point data should contain all mature cell types. Therefore, we intrinsically selected the end-point data that has 8 lineage types and 30 cell types as a reference to identify both lineage and cell-type markers. In total, the 2 independent studies cover 30 cell types along the 13 developmental stages. Next, we transformed the combined gene expression matrix into a 30-cell-type score matrix and built an integrative lineage map of the developing zebrafish embryo. Because of the design differences, we manually summarized all developmental stages into 13 major stages (Fig. 1b). Instead of assigning cells to these 30 cell types, we annotated them as eight major lineage types using our previously published method MACA^20^. Briefly, MACA is a marker-based cell-type annotation tool that searches consensus between cluster-level and cell-level labels. Due to the lack of a marker database for zebrafish embryos, we used the marker identification function in MASI to identify lineage-type marker genes. Then, we can visualize how lineage compositions change along the developmental timeline (Fig. 1c). First, we found that the two studies are largely consistent. Second, we observed a decline of germline and lineage diversification along these developmental stages (Fig. 1c). We further investigated the original time point of different cell lineages based on our integrated lineage map by visualizing cell-type score (Supplementary Fig. 3). We found that the development of germline can be retrieved back at least at the 3 hpf time-point (Fig. 1d). In Wagner et al., the earliest time point at which germline cells were observed is 4 hpf. However, in Farrell et al., authors report that the germ layer appears before 4 hpf and that many other lineages do not separate until 4 hpf. This is consistent with our finding from the integrated lineage map, where we show that germline cells are observed at 3 hpf and are the major cell lineage composition until that time point (Fig. 1c and d). The notochord defines the longitudinal axis of the embryo and determines the orientation of the vertebral column, and our analysis suggests the notochord emerges at around 7 hpf, while both Farrell et al. and Wagner et al. showed the emergence of the notochord takes place between 6hpf and 8hpf. We also observed that epidermal lineage appears at 3 hpf (Fig. 1c), consistent with Farrell et al. who observed this epidermal lineage at 3.3 hpf. Additionally, we observe that non-neural ectoderm separates from epidermal cells at 12 hpf in our analysis, as seen in Farrell et al.^46^. Taken together, these three analyses with temporal datasets demonstrate the power of cell-type score matrix in the simple and intuitive approach for integrative lineage analysis.

### Workflow of MASI for integrative analysis

**Fig. 2:**
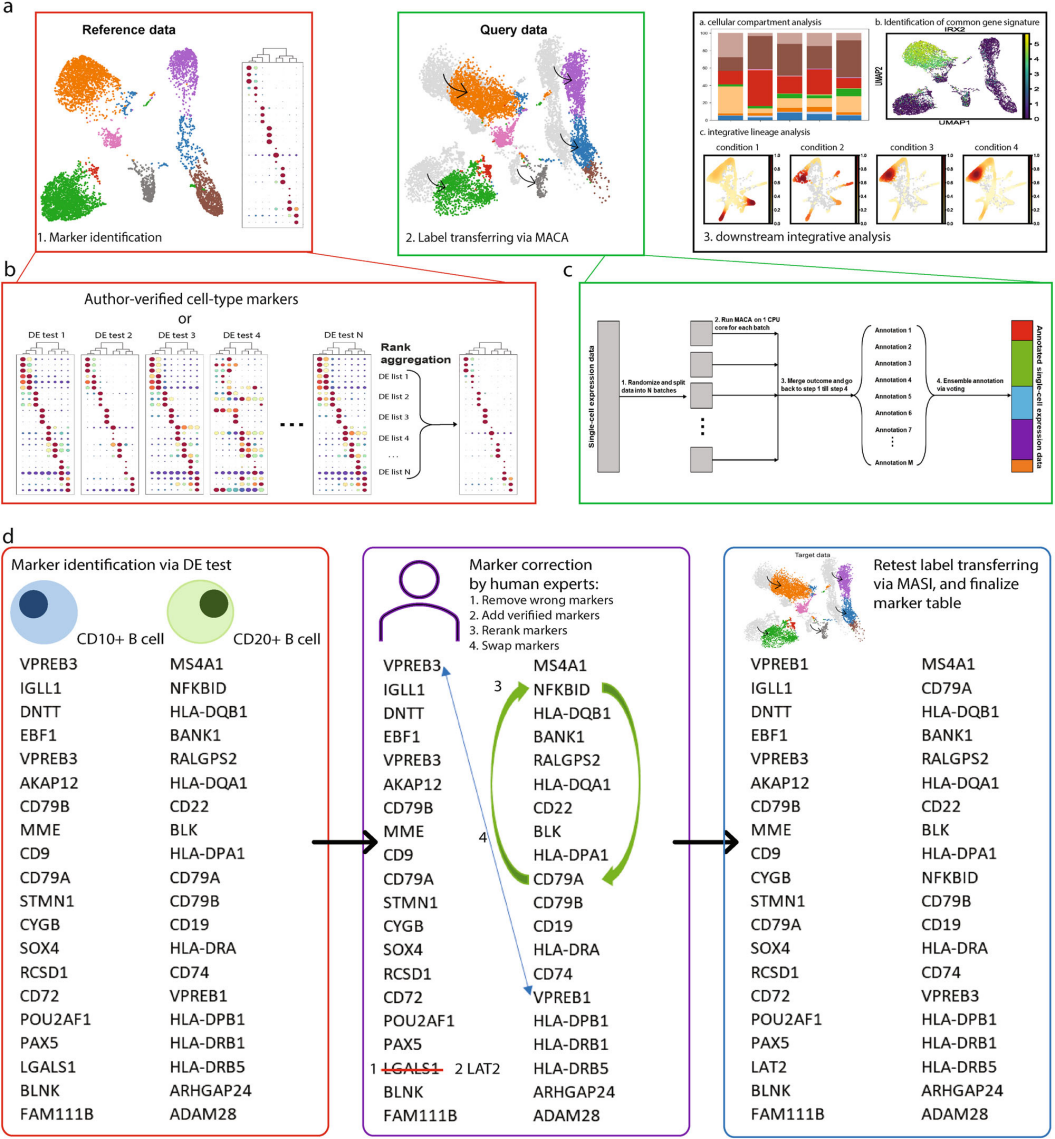
Integrative annotation pipeline through MASI. **a** A workflow of integrative annotation through MASI, including marker identification from reference data, label transferring by MACA, and downstream integrative analyses. **b** Ensemble approach to identify robust cell-type markers from reference data. N DE test outcomes are aggregated to get the final ranked marker list. **c** Parallel computation for fast annotation in order to accommodate large-scale scRNA-seq data. **d** Suggested actions for improvement of label transferring. Human experts can correct wrong markers, adjust marker ranking, and so on, in order to improve annotation accuracy by MASI.

Having demonstrated the advantages of a cell-type score matrix and chosen a suitable processing pipeline, we next describe the full MASI workflow to annotate and integrate query data based on fully annotated reference data. MASI first identifies cell-type marker genes from the reference data (Step 1), then processes data with pipeline #14 (Step 2), next annotates cell types via MACA^20^ (Step 3) and performs other downstream integrative analyses (Step 4) (Fig. 2a). The first step of the MASI workflow is to identify marker genes for each cell type via differential expression (DE) tests if author-verified markers are not available. To select the DE method that can facilitate accurate cell-type annotation through MACA, we benchmarked 12 DE tests, including common DE tests implemented in Scanpy^47^ and Seurat^7^, and two newly proposed methods COSG^48^ and Cepo^49^. For the 6 benchmark datasets, we found that marker genes obtained from these 12 DE tests have varying performances in terms of predicting cell types using MACA (Supplementary Fig. 4). This is consistent with results shown in other benchmark studies on DE tests^50–52^, where no single DE test can faithfully identify reliable cell-type markers for all single-cell data. To account for the influence of single DE tests, we decided to construct ranked cell-type markers via an ensemble approach (Fig. 2b and marker rank aggregation in the “Methods” section)^53^. In Step 2, we process reference and all query datasets following pipeline #14, since we have shown that pipeline #14 can remove batch effects in most cases of scRNA-seq integration. At this step, MASI will return a cell-type score matrix. This is a very critical component for downstream annotation and other integrative analyses. Next (Step 3), we annotate all datasets through MACA. However, the MACA algorithm wasn’t initially designed to handle large-scale scRNA-seq. To accommodate large-scale scRNA-seq data, we refactored the MACA annotation workflow in a parallel manner by splitting data into multiple batches and distributing annotation onto multiple CPU cores (Fig. 2c). This enables MACA to perform integrative analysis for large-scale scRNA-seq with limited computational resources while not losing annotation accuracy. Finally in Step 4, users can use the returned cell-type score matrix and cell-type annotation to do other downstream integrative analyses based on their own needs.

### Building a marker collection for standardized cell-type annotations

Using the ensemble approach (Fig. 2b) to automatically identify markers for cell types across species and tissues, we built a marker collection for cell-type annotation. In addition, we also added author-verified marker tables into our collection where available. This marker collection is deposited at the MASI GitHub. Of note, our marker collection is customizable, where users can add, delete, or readjust marker gene ranking (Fig. 2d). Meanwhile, presenting cell-type markers in tables enables more flexibility to assemble cell-type markers from multiple references. For example, users can concatenate columns of new cell types from additional references into the existing cell-type marker table. With this marker gene collection, we could apply MASI for integrative analysis of scRNA-seq datasets in different scenarios. In the following sections, we benchmarked MASI with other well-established methods for the task of cell-type annotation and data integration in terms of reliability, speed, and accuracy. Finally, we provided case examples in three different scenarios.

### Benchmarking cell-type annotation and data integration

In our benchmark of MASI with other well-established methods, we used the same six mixed-batch datasets above. We selected linear and non-linear support vector machine (SVM) classifiers as supervised methods, as another benchmark study has demonstrated that SVM outperformed other sophisticated cell-type annotation methods^54^. scNym^10^ and scArches^11^ are semi-supervised deep learning methods for cell-type annotation and data integration, and we included these two methods in our benchmark. A new efficient data integration tool, Symphony, demonstrated the great power of label transferring for large-scale scRNA-seq data^17^. To integrate scRNA-seq data, Symphony is built upon an extensively examined integration method, Harmony^5^. So, we also included it in our benchmark. Besides these label transferring methods, we further included batch-effect correction methods, including Scanorama^16^, LIGER^6^, and Seurat^7^, since these three batch-correction methods were listed as top methods by a previous benchmark study^55^. After removing batch effects with these three methods, we trained the *k*-nearest-neighbors classifier to transfer labels from reference to query data. For a fair comparison, our benchmark study was performed on a local workstation with 64GB memory and Nvidia Quadro RTX 6000 as GPU support. Of note, both scNym and scArches use GPU to speed up computation, while other methods will not use GPU for computing. Meanwhile, both LIGER and Seurat heavily rely on computing memory, and we were unable to perform these two methods if computation exceeds the memory limit.

**Fig. 3:**
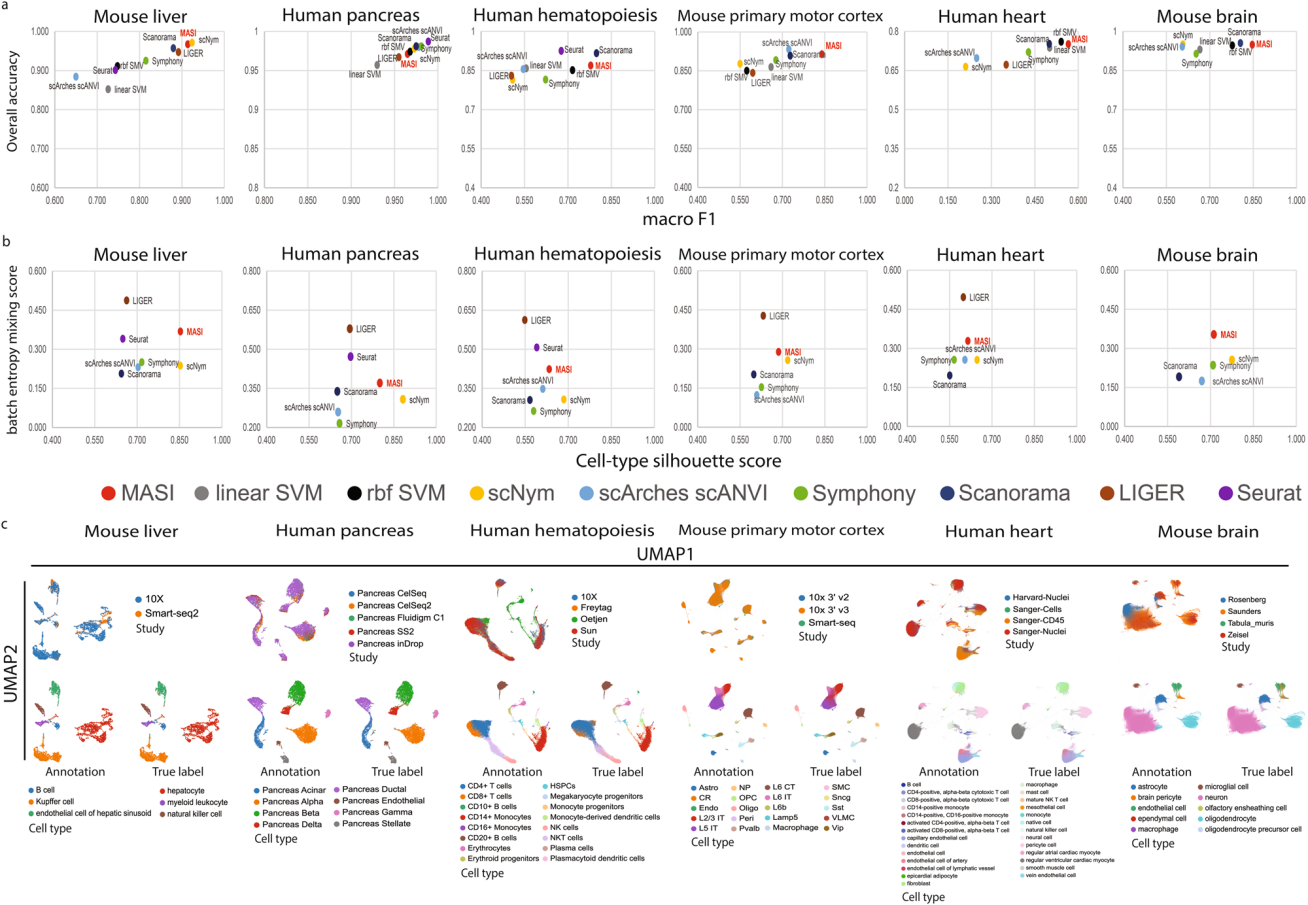
Batch correction and label transferring benchmarks. **a** Comparison of label transferring for MASI, supervised, and semi-supervised methods. ACC: overall accuracy. Macro F1 is the average of F1 scores per cell type. A higher score in both metrics suggests better cell-type prediction. **b** Comparison of batch correction for MASI, scNym^10^, scArches scANVI^11^, Symphony^17^, Scanorama^16^, LIGER^6^ and Seurat^7^. Cell-type silhouette score measures how well the integrated representation by these methods preserves cell-type variation, while the batch entropy mixing score measures how well the same cell type from different batches is mixed. **c** Visualization of MASI integration through UMAP. Cells are colored according to MASI-reported annotation (top left), author-reported annotation (top right), and batch id (bottom left).

We first focused on how well transferring cell-type labels from reference data to query data is done by these methods. We intentionally selected datasets that have the greatest number of cell types as references. So, methods will see all possible cell types during training. We used macro F1 and overall accuracy to quantify the performance of these methods in terms of how accurate annotation is for each cell type and how accurate annotation is for the overall dataset. We found that all methods have similar performance in terms of overall accuracy, but MASI showed consistently higher macro F1 scores across all benchmark datasets, indicating balanced annotation across major and minor cell types (Fig. 3a). In the human heart atlas, the authors provided two levels of annotation. A high hierarchy annotation consists of 9 major cell types in the human heart, and the low hierarchy annotation consists of 27 refined subtypes deriving from those 9 major cell types^12^. When reference data is organized in such a hierarchical structure, it is advantageous to annotate query data in such a way. Thus, we transferred cell-type labels at 2 annotation levels with all 9 methods. We found all methods demonstrated decent accuracy for high hierarchy annotation, but they deteriorate when annotation reaches low hierarchy, except MASI, SVM, Symphony, and Scanorama (Supplementary Data 2). Results above suggest an advantage of MASI in (1) annotating non-major cell types, considering most single-cell data are class imbalanced and (2) annotating single-cell data in a hierarchical structure.

Next, we evaluated how well the integrated representations learned by these methods capture the cell-type structure while mixing batches, using the cell-type silhouette score and batch entropy mixing score mentioned above. Though LIGER always achieved the highest batch entropy mixing score, it doesn’t necessarily present the correct cell type structure, for example, the integration of human hematopoiesis data by LIGER. Cell types, like Erythroid progenitors only presenting in Oetjen et al., were mixed with other cell types (Supplementary Fig. 5). Again, MASI demonstrated a good balance between capturing cell-type variation and batch mixing (Fig. 3b). We found all seven methods, MASI, scNym, scArches, Symphony, Scanorama, LIGER, and Seurat, achieved the same purpose of mixing data from diverse sources in human pancreas and human hematopoiesis data (Fig. 3c and Supplementary Fig. 5). However, we observed that representations learned by scNym, scArches, Symphony, Scanorma, and Seurat captured weaker correlations among different cell-types, while the cell-type score representation of MASI and representation of LIGER preserved distinct cellular correlation, especially in human hematopoiesis (Supplementary Figs. 6 and 7). As we showed in integrative lineage analysis, the cell-type score matrix does not only capture the biological transition but also reserves a stronger cellular correlation. Taken together, we conclude that integration by MASI preserves meaningful biological information, over other integration methods.

To give a quantitative performance report across all six benchmark datasets, we summed the cell-type silhouette score and the batch mixing entropy score to obtain the integration score, and we summed the macro F1 and the overall accuracy to generate the annotation score. The overall performance score was then calculated as an average of the two scores. For annotation, paired *t*-tests showed that MASI is significantly better than other methods (*p*-values < 0.05) except Scanorama and Seurat. For integration, MASI has significantly higher scores than scArches scANVI, Symphony, and Scanorama (*p*-values < 0.05). Overall, MASI outperforms all other methods (*p*-values < 0.05) except Seurat (Supplementary Data 3). Over all six benchmark datasets, MASI was also ranked as one of the top methods for both data integration and annotation (Supplementary Data 3).

### Dependence on choice of reference dataset and clustering resolution

Like all other reference-based label-transferring methods, MASI is highly dependent on reference data. Therefore, MASI will not be able to annotate cell types in query data that have not been seen in reference data. However, it is still worth answering if a cell-type score matrix constructed with reference data with less cell types can preserve the cell-type structure for query data that contains extra unseen cell types. To understand the impact of choice of reference dataset on the cell-type annotation in the query dataset, we swapped reference data from Oetjen et al. to 10x Genomics data in the human hematopoietic benchmark dataset. The 10x Genomics data has only 12 cell types, while Oetjen et al. identified 16 cell types in their original report. Thus, the query data would contain 4 extra unseen cell types. We performed marker gene identification and transformed the gene expression matrix to cell-type score matrix using 10x Genomics data as a reference. We observed that the 12-dimension cell-type score matrix built upon the 10x Genomics dataset as a reference can reveal cell-type structure for the Oetjen et al. data that had 16 author-reported major cell types in total (Supplementary Fig. 8)^27^. However, as erythrocytes and erythroid progenitor cell-types are not present in the reference, MASI mislabeled them as CD14+ monocytes and HSPCs, respectively (Supplementary Fig. 8). We next asked if we could identify subtypes from MASI-reported cell types to match the author-reported annotation resolution. Here, we used SCCAF, a computational method that was previously proposed for the identification of putative cell types through a machine learning approach^56^. The concept behind this machine learning is: if the clustering resolution reflects the number of true cell types within the data, a machine learning classifier can achieve high accuracy with the clustering label. Thus, we applied SCCAF to identify potential subtypes for each major cell type identified by MASI. We evaluated how well these three approaches, MASI annotation alone, SCCAF identification alone, and SCCAF+MASI annotation combined respectively, could reveal a similar annotation resolution to the author’s annotation by calculating ARI and NMI. We found that SCCAF+MASI annotation matches the author’s annotation resolution more than MASI annotation and SCCAF identification alone (Supplementary Fig. 8).

Besides combining MASI and SCCAF for refining annotation, an alternative solution to annotate unseen cell types in query data would be treating them as unassigned. In order to identify these unseen cell types, we designed the certainty score (see certainty score in the “Methods” section). If the certainty score of MAIS-reported annotation is lower than a threshold, we could report unassigned instead of giving the cell a definite cell-type label. Next, we examined how well we can retrieve those 4 extra cell types in Oetjen et al. by thresholding the certainty score. We found setting the threshold of certainty score between 0.5 and 0.6 would retrieve these 4 extra cell types with decent accuracy (Supplementary Fig. 9). Using either a low or high threshold would either retrieve less unseen cell types or mark the majority of cells as unassigned.

To summarize cell-type identification, we conclude that the choice of reference data is critical to the performance of MASI. We encourage users to search for the most comprehensively annotated reference data if available. Though the criteria for the selection of reference data can vary across users and it depends on specific research needs, here are two suggestions we have. First, a quality reference could contain multiple annotation resolutions, from high to low, like the human heart atlas data^12^. Second, the reference should also contain enough cells for each cell type, in order to call out reliable marker genes through DE tests. Even so, reference may not help users get annotation resolution as desired. To unravel potential subtypes, users can either combine SCCAF and MASI to reach a finer annotation or use the certainty score to identify unseen cell types.

### Annotation of spatial transcriptomics data with MASI

Next, we used MASI to map cell type labels from scRNA-seq data to sequencing-based spatial transcriptomics data. We tested this idea on spatial hippocampus data profiled by Slide-seqV2, since Slide-seqV2 reaches a higher resolution of spatial profiling than 10X Visium^57^. Integrating Slide-seqV2 with scRNA-seq further suggests a potential application of MASI in spatial transcriptomic analysis (Supplementary Fig. 10a). MASI was able to assign cell type labels to the mouse hippocampus Slide-seqV2 data (Supplementary Fig. 10b). Spatial expression patterns of marker genes for 5 distinct cell types also match with their cell locations in space (Supplementary Fig. 10b–d).

### Case studies to explore data integration and standardization in large datasets

In the last three sections, we applied MASI to three case studies consisting of 11,3018, 251,057, and 1,196,523 cells from kidney, COVID-19 datasets, and heart. These datasets consist of 27, 57, and 27 cell types, respectively.

#### Case 1: Using human kidney atlas for integration of single-cell human kidney across multiple conditions

**Fig. 4:**
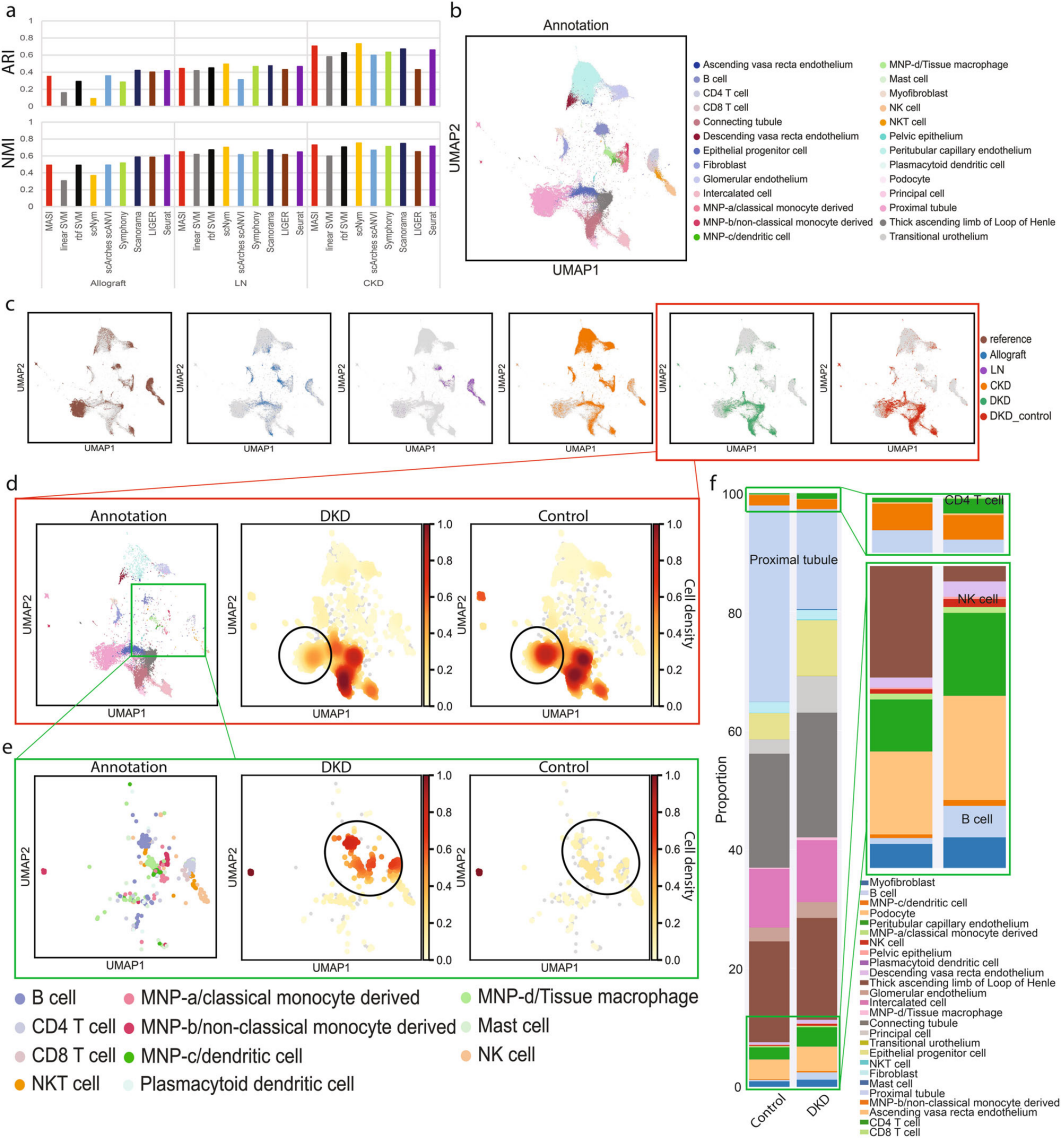
Transferring human kidney atlas for integration of single-cell human kidney across conditions. **a** Comparison of label transferring for MASI, supervised, and semi-supervised methods. ARI and NMI are calculated by comparing method-reported annotation with author-reported annotation in a study-wise manner. **b** Visualization of the integrative annotation by MASI. Cells are colored according to MASI-reported cell-type annotation. **c** Visualization of integration by MASI. Cells are colored according to the study id. **d** and **e** Population densities in DKD and control samples. Cell type annotation is shown on the left panel. Cell-type population densities of DKD and control samples are presented separately to highlight differences in cell-type populations. **f** Quantitative measurement of cellular compositions. CD4+ T cell, NK cell, and B cell are zoomed in to show the difference between control and DKD groups.

The first human kidney atlas profiled 27 distinct cell types in a mature kidney, containing 25,128 cells^58^. This atlas provides a good reference to study cellular irregularities in kidney diseases. So far, independent single-cell studies have been conducted to reveal mechanisms in different kidney diseases^59–62^. An approach that can provide an integrative view of multiple kidney diseases may further add insight into how cellular irregularities vary among different kidney diseases. We used kidney atlas data as a reference and mapped cell-type labels to human kidney data that were collected under different conditions, including allograft kidney (4487 cells), LN (lupus nephritis, 2838 cells) CKD (chronic kidney disease, 51,849 cells) and DKD (diabetic kidney disease, 28716 cells with internal control). Because cell-type naming and annotation resolution vary among these studies, we changed to use ARI and NMI for evaluation. Benchmarking in this task showed MASI has better agreement with author-reported annotations with consistency (NMI values of 0.49, 0.648, and 0.728, respectively) (Fig. 4a). Overall mapping, cell type standardization, and batch-mixing results are shown in Fig. 4b and c. Next, we focused on the human DKD data, which came with its control set. The population density map suggested a decrease of the proximal tubule (Fig. 4d) and an increase of immune cells (Fig. 4e), especially an increased cellular composition of NK cell, B cell, and CD4+ T cell (Fig. 4f). This is consistent with an increase of immune response identified in DKD^60^.

#### Case 2: transferring human lung atlas for integration of single-cell COVID-19 data across participants

**Fig. 5:**
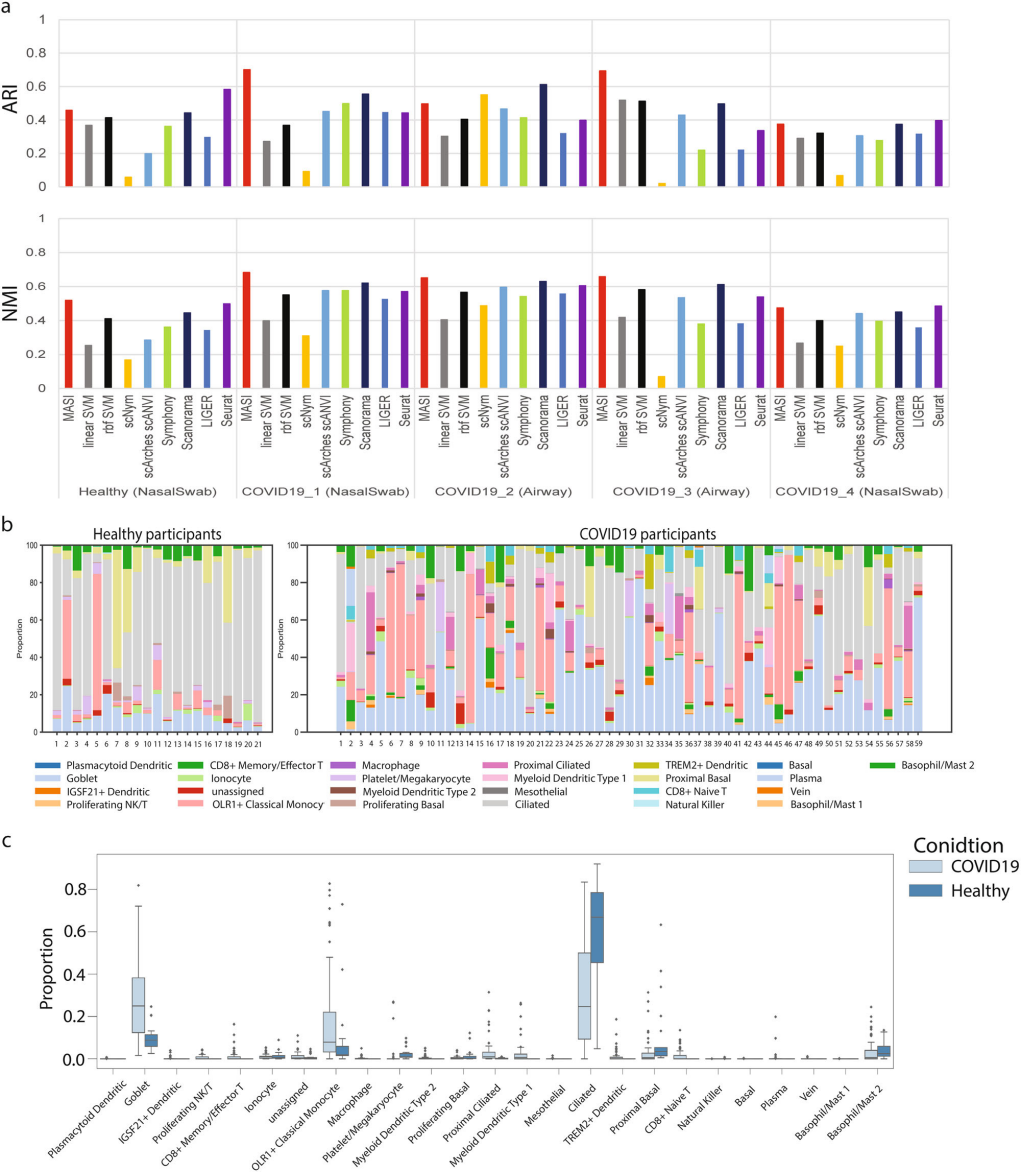
Transferring human lung atlas for integration of single-cell COVID-19 data across individuals. **a** Comparison of label transferring for MASI, supervised, and semi-supervised methods. ARI and NMI are calculated by comparing method-reported annotation with author-reported annotation in a study-wise manner. **b** Cellular compositions of healthy and COVID-19 participants^14,15^. Each column represents one individual. **c** Cellular composition comparison of healthy and COVID-19 participants. A box covers 25th percentile to 75th percentile samples. Within a box, a median line was drawn. Bottom and top error bar lines define 5-percentile and 95-percentile.

Our second MASI application is transferring knowledge learned from the human lung atlas to understand the global COVID-19 pandemic at the cellular level among healthy and COVID-19 participants. The human lung atlas data (75,071 cells) served as reference data with 57 identified subtypes^63^. Using this annotation, we aimed to annotate 80 COVID-19 samples collected from nasal swabs (58 participants) and airways (22 participants) across different individuals, with 175,986 cells in total^14,15^. These COVID-19 data included negative (21 participants) and positive samples (59 participants) from multiple centers. Due to cell-type annotation and resolution differences, we cannot directly compare cellular differences between healthy and COVID-19 participants. We used MASI to annotate the COVID-19 data to match the annotation resolution of the human lung atlas. Again, we benchmarked MASI with two SVM classifiers, scNym, scArches, Symphony, Scanorama, LIGER, and Seurat, using ARI and NMI as evaluation metrics. We found MASI shows consistently great agreement with author-reported annotations for all COVID-19 data compared to the other 8 methods (Fig. 5a). Since cell-type annotations for all participants were leveled up to the same resolution, we were able to directly compare the cellular differences between healthy and COVID-19 participants (Supplementary Fig. 11). We observed distinct cellular compositions between healthy and COVID-19 groups, and the distinct cellular composition is consistent across participants within the same group (Fig. 5b). Then, we quantified the changes of cellular composition for all cell types and found an increase in the proportion of Goblet cells and a decrease in ciliated cell proportions in the COVID-19 group (Fig. 5c). This discovery may explain other investigations of SARS-CoV-2 virus targeting ciliated cells via *ACE2*^64,65^.

#### Case 3: Cell-type annotation and batch-mixing of human heart datasets

**Fig. 6:**
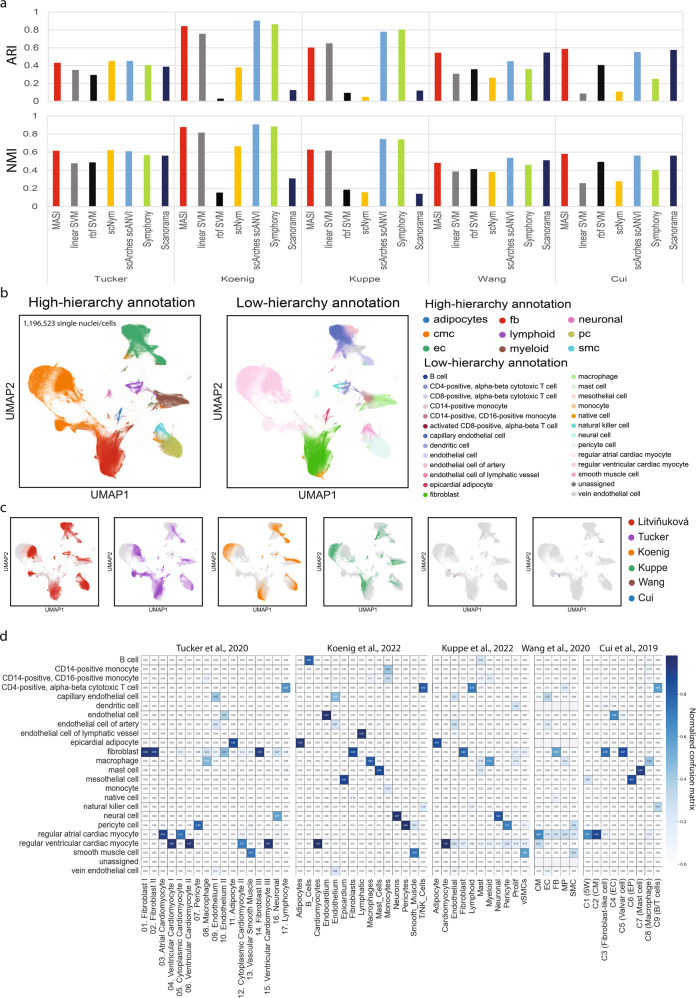
Transferring human heart atlas for integration of single-cell human heart across research groups. **a** Comparison of label transferring for MASI, supervised, and semi-supervised methods. ARI and NMI are calculated by comparing method-reported annotation with author-reported annotation in a study-wise manner. **b** Visualization of the integrative annotation by MASI. Cells are colored according to MASI-reported cell-type annotation at both high and low hierarchy levels. **c** Visualization of integration by MASI. Cells are colored according to the study id. **d** confusion matrix of MASI-reported annotation against author-reported annotation. Confusion matrix is normalized to have column sum as 1. Row names use the naming style of human heart atlas, and column names remain the original naming styles of Tucker et al.^13^, Koenig et al.^66^, Kuppe et al.^67^, Wang et al.^69^, and Cui et al.^68^ data.

Tucker et al.^13^ and human heart atlas (Litviňuková et al.)^12^ provide two atlas-level resources for human health heart data at single-cell resolution. More recently, two studies by Koenig et al. and Kuppe et al. profiled atlas-scale single nuclei/cell transcriptome of heart failure and myocardial infarction, respectively^66,67^. In addition, other human heart datasets are also available^68,69^. However, these studies did not use the same cell-type naming style, and they reported annotations at different resolutions. The human heart atlas with 486134 cells in total identified 27 subtypes while Tucker et al. (17 subtypes, 287,269 cells), Koenig et al. (15 subtypes, 220,752 cells), Kuppe et al. (11 cell types, 191,795 cells), Wang et al. (5 cell types, 6731 cells), and Cui et al. (9 cell types, 3842 cells) reported different numbers of cell-types in their own studies^12,13,66–69^. We think uniform annotation of cell-type labels and batch-mixing of these datasets can yield insights into common themes and inter-human variability across these datasets. Since the human heart atlas data provided both high and low hierarchy annotations and its low hierarchy annotation revealed the greatest number of subtypes, we chose human heart atlas data as the reference. Because the data size exceeds our computation capacity, we were unable to run LIGER and Seurat for the integration of human heart datasets. For all 7 methods compared here, we found they have similar performance for mapping cell-type labels to Tucker et al., but MASI, scArches, and Scanorama show better outcomes than the other 4 methods in both Wang et al. and Cui et al. (Fig. 6a). Surprisingly, the non-linear SVM had a worse annotation for Koenig et al. and Kuppe et al., compared to its linear counterpart. Methods, including scNym and Scanorama, also showed disappointing annotation outcomes in these two datasets (Fig. 6a). Benchmarking in human heart datasets again demonstrated that MASI has consistent annotation performance. Relying on a greater resolution of Litviňuková et al. data, we were able to standardize annotation for the other five studies at two levels, high and low hierarchy, respectively (Fig. 6b). We visualized integration via MASI and observed no distinct batch differences (Fig. 6c). We noticed MASI annotated a number of cells in Tucker et al. as fibroblast while the author-reported annotation for these cells includes cardiomyocyte, endothelium, and neural cells (Fig. 6d). We looked deeper into these cells and examined expressions of marker genes for fibroblast, endothelium, and neural cells and found that the disagreement between MASI-reported and author-reported annotation is likely due to background mRNA from fibroblasts (Supplementary Fig. 12). With MASI, we identified pericyte in Wang et al. and natural killer cell in Cui et al., which were not reported by the authors (Fig. 6d and Supplementary Fig. 13).

### MASI is fast and can accommodate annotation for large-scale single-cell data

Taken together, we show that MASI can quickly annotate large-scale scRNA-seq data. Our runtime test showed runtime of model-based methods increases dramatically once the data scales up (Supplementary Fig. 14). For an extreme test, we performed integration of mouse brain data (nearly 1 million cells) by MASI on a personal laptop, with 16 GB memory and no GPU support. Without sacrificing annotation accuracy, MASI can scale up to accommodate label transferring for 1 million cells (Supplementary Data 4).

## Discussion

Here, we present MASI, a new tool to quickly and accurately annotate single-cell datasets based on marker genes obtained from a reference dataset. We show that MASI can also be used for batch-mixing and serve as a data integration method for single-cell transcriptomics data. We benchmarked MASI with supervised and semi-supervised methods, and our results show that the performance of MASI is comparable or even superior to other tested methods based on the datasets used in this study. A core component of MASI is the conversion to a cell-type score matrix. We have shown that cell-type scores can be used as features for integrative lineage analysis and demonstrated their intuitive interpretability. Finally, we showed the utility of MASI in three different case studies of data integration covering different biological conditions, surveyed participants, and research groups. Like other supervised and semi-supervised methods that rely on reference data, accurate annotation via MASI is also dependent on the quality of reference data. Thus, the choice and resolution of the reference are critical to downstream analysis. We would recommend users to select reference data that provides annotation resolution compatible with their downstream investigations. If query data has unseen cell types not in reference, MASI in combination with SCCAF can be used to identify subtypes within major cell types. Additionally, we showed that MASI can also be applied for cell-type prediction in spatial transcriptomics datasets using comparable single-cell transcriptomics datasets as a reference.

There are many well-established integration methods available to address batch effects in scRNA-seq datasets, for example, Seurat, Harmony, and LIGER^5–7^. Additionally, some deep learning-based methods such as HDMC and CarDEC are also available^70,71^. In this study, we rigorously tested cell-type score-based integration via MASI across various single-cell platforms, cytoplasm/nuclei, research groups, conditions, and individuals. Our analyses suggest that marker-based feature engineering can be useful for reference-based cell-type annotation, batch-mixing, and data integration.

Overall, MASI is easy to set up and requires limited computation resources to run. It can be used for reference-based cell-type annotation and batch-mixing, which could facilitate quick hypothesis-driven exploration of diverse datasets obtained from different labs. Moreover, the democratization of single-cell transcriptomics data (larger cellular output with lower cost) could empower researchers even with limited computational resources to investigate millions of single cells among diverse biological systems.

## Methods

### Data preprocessing

Raw gene expression counts data were ‘LogNormalized’, which divides the total count in that cell and multiplies it by a scale factor of 10,000 (in all our analyses), followed by log-transformation to get the normalized expression matrix. For implementing MASI, we skipped the step of calling highly variable genes, because only the identified marker genes were used for integrative annotation. For training scNym and scArches, we used the top 5000 highly variable genes by batch, which were calculated using the function “pp.highly_variable_genes” in Scanpy^47^.

### Marker rank aggregation

We considered two ensemble marker ranking schemes. In the first scheme, the top 20 marker genes from each DE test were compiled together. For the second scheme, only statistically significant marker genes based on the *p*-values corrected for multiple hypothesis correction were considered. In the first scheme, we searched the consensus ranking via robust rank aggregation^53^. In the second scheme, rank aggregation was done through Lancaster combination^72^.

### Weighing markers

When data to be annotated contains distinct cell types and cell types do not share marker genes, we reasoned that weighing markers would not influence the final annotation by MASI. However, this can be beneficial to distinguish cell subtypes that share common markers, for example, subtype T cells. We used a simple weighing strategy that returned good label transferring. Given *N* markers for cell type *A*, the 1st marker in this ranked list will contribute 100% of its expression to the cell-type score of *A*, while the *N*th marker only contributes 50% of its expression. For the *i*th marker in the rest, we form this discount calculation as (1) $1-(\frac{i}{N})\,\ast \,(\frac{1}{2})$ to get their weights in cell-type *A*. Beside the weighing strategy above, other weighing strategies, including Rank Order Centroid and Ratio method, can also be considered for customization.

### Converting gene expression matrix to cell-type score matrix

Cell-type score for a given cell-type A with N expressed markers is calculated by summing up the expression of all *N* markers with consideration of weighing markers as above. This is defined as the raw cell-type score. From this, the PlinerScore is calculated by adding a TF-IDF transformation and suppressing expression values of a marker gene to zeros if they are below the X-percentile of expression values across all cells before the raw cell-type score conversion. The default value for PlinerScore threshold is 0.25 as the percentile threshold^18^.

### Classification by linear and non-linear SVM

Both linear and non-linear SVM classifiers can be impacted by feature selection. As a benchmark reported, linear and non-linear SVM can have varying prediction accuracies for scRNA-seq data when different feature selection processes were applied^73^. Nevertheless, using more discriminative features should improve the accuracy of these two supervised models. Instead of using highly variable genes and PCA-reduced features, we used the same cell-type markers that were used for MASI to train both linear and non-linear SVM classifiers. This is primarily because we observed cell-type markers have a good balance between cell-type preservation and batch-effect removal, compared to both highly variable genes and PCA-reduced features, as shown in Supplementary Fig. 1.

### Label transferring through MASI

Once cell-type markers are identified, Mapping cell-type labels to query data is performed using MACA^20^. Briefly, for each cell, MACA generates two labels: the per-cell cell-type Label 1 and group-based clustering Label 2. Then, MACA maps clustering Label 2 to cell-type Label 1 to get the overall cell-type annotation. In MACA, we used different clustering parameters to generate multiple Label 2s, for the purpose of reproducibility^20^. In this study, we also ran Louvain community detection with a range of clustering parameters to get multiple clustering Label 2s. These include clustering resolution 3, 5, 7 with 5, 10, 15 as neighborhood sizes to over-cluster cells. With multiple clustering Label 2s, we were able to map them to Label 1 and get a more reproducible ensembled cell-type annotation. To accommodate for large-scale scRNA-seq data, we split the whole data into N batches and ran MACA with one batch per CPU core.

### Label transferring through scNym and scArches

Both scNym and scArches are deep-learning-based transfer learning methods. Therefore, an optimal outcome for a specific data might require customized parameter tuning. However, for benchmarking, we used default pipelines of both methods for all data involved in this study. Respective tutorials can be found at https://github.com/calico/scnym and https://scarches.readthedocs.io/en/latest/scanvi_surgery_pipeline.html.

### Label transferring through Symphony

A developer-provided tutorial on using Symphony could be found on GitHub (https://github.com/immunogenomics/symphony). In this study, we first built a reference with genes by cell matrix. Multiple procedures, including variable gene selection, scaling, PCA, and batch correction via Harmony, were already implemented in Symphony. Next, we mapped cell-type labels from reference to query data and integrate query data with reference data at the same time, using Symphony. The default tutorial for using Symphony can be found at https://github.com/immunogenomics/symphony.

### Label transferring using Scanorama, LIGER, and Seurat

All Scanorama, LIGER, and Seurat are primarily batch-correction methods. Thus, we first followed their own tutorials to remove batch effects. Then, we used the batch-corrected latent space to train *k*-nearest neighbors classifiers, with *k* as 5 all datasets.

### Certainty score

We designed a certainty score to quantify how certain the assigned cell-type annotation for a cell. The certainty score is defined as (2) $C=1-\frac{{D}_{1{\rm {st}}}}{{D}_{N{\rm {th}}}}$, where *D*_1st_ is the distance to the closest cell-type centroid *D*_*N*th_ is the distance to least close cell-type centroid in reference. The intuition behind is that if it is certain that a cell belongs to cell type A, the cell would have small *D*_1st_ to the centroid of cell type A and small *D*_*N*th_ to the centroid of an unrelated cell type. Therefore, $\frac{{D}_{1{\rm {st}}}}{{D}_{N{\rm {th}}}}$ would be small, and *C* would be closer to 1.

### 2D visualization using UMAP

To visualize integrations by these three methods, we used the same parameter setting for all datasets. We set up metrics “cosine” to define distance, cells within 0.1 were considered as neighbors, and minimum of 15 cells form a community.

### Integrative lineage analysis

We used ForceAtlas2 with PAGA (partition-based graph abstraction) initialization to layout integrative lineage maps with cell-type scores instead of any other hidden space features, like principal component analysis (PCA) representation or representation from neural network model^74,75^. To initialize PAGA, we performed Louvain community detection to assign cells as multiple meta cells^76^. We used resolution 5 for Louvain community detection in order to get enough meta cells. Once cells are laid out on the ForceAtlas2 space, we directly visualize lineage paths with cell-type scores, without clustering cells into cell types.

### Evaluation metrics

(3) Overall accuracy: $${{{{{\rm{Acc}}}}}}=\frac{{{{{\rm{{Total}}}}}}\,{{{{{\rm{number}}}}}}\,{{{{{\rm{of}}}}}}\,{{{{{\rm{correction}}}}}}\,{{{{{\rm{predictions}}}}}}}{{{{{{\rm{Total}}}}}}\,{{{{{\rm{number}}}}}}\,{{{{{\rm{of}}}}}}\,{{{{{\rm{cells}}}}}}}$$.

(4) Macro *F*1: $F1=\frac{\rm {{precision}}\,\ast \,{\rm {recall}}}{({\rm {precision+recall}})}\,\ast \,2$. *F*1 was calculated for each cell type, then ${\rm {macro}}\,F1$ is the average of *F*1 scores for all cell types. Because this metric doesn’t consider class weights for imbalanced data, a higher ${\rm {macro}}\,F1$ could suggest correction predictions for both dominant and non-dominant cell types.

Cell-type silhouette score: We first used function “sklearn.metrics.silhouette_score” in scikit-learn Python package to calculate a typical silhouette score *S*^77^. The author-reported cell type label served as the ground truth. This calculation uses the hidden space returned by integration methods with cell-type labels. Both scNym and scArches learned a 10-dimension hidden space representation by default. The lower representation by MASI depends on the number of unique cell types available in the reference dataset. Next, we re-scaled the score from 0 to 1 by (1+*S*)/2, defined as a cell-type silhouette score. The higher the score is, the better cell-type variation is captured.

(5) Batch entropy mixing score^78^: $E={\sum }_{i=1}^{c}{x}_{i}\,\log ({x}_{i})$. In this study, *x*_*i*_ is the proportion of cells from batch *i* in a region of the first two UMAPs, and ${\sum }_{i=1}^{c}{x}_{i}=1$. This score should quantify how well-mixed cells from different batches are in a region. The same as *Cell-type silhouette score*, the calculation of *Batch entropy mixing score* is based on the hidden space returned by integration methods with batch information as label. The higher the score is, the better mixing.

*Adjusted rand index (ARI)*: The rand index (RI) measures a similarity or agreement between two clustering labels. The ARI then is defined through (6) ${{{{{\rm{ARI}}}}}}=\frac{{{{{{\rm{RI}}}}}}-{{{{{\rm{expected}}}}}}\,{{{{{\rm{RI}}}}}}}{\max ({{{{{\rm{RI}}}}}})-{{{{{\rm{expected}}}}}}\,{{{{{\rm{RI}}}}}}}$. In this study, we used ARI to measure the agreement between cell-type annotation reported by a transfer learning method and the author-reported cell-type annotation.

*Normalized mutual information (NMI)*: Like ARI, NMI also qualifies the agreement between two clustering labels. It is defined as (7) ${\rm {NMI}}=\frac{I(P,T)}{\sqrt{H(P)H(T)}}$. *P* and *T* are empirical categorical distributions for the predicted and real clustering, *I* is mutual entropy, and *H* is the Shannon entropy.

### Score aggregation and ranking

To report aggregated scores and rank the data processing pipelines and integration methods benchmarked in this study, we selected a weighted averaging approach to quantify our performances in the tasks of integration and annotation. This is similar to the weighted averaging in the benchmark study by Luecken et al.^79^. The integration score is defined as (8) ${S}_{{\rm {integration}}}=0.7\times {S}_{{\rm {cell-type}}}+0.3\times {S}_{{\rm {batch-mixing}}}$, while the annotation score is defined as (9) ${S}_{{\rm {annotation}}}=0.7\times F{1}_{{\rm {macro}}}+0.3\,\times AC{C}_{{\rm {overall}}}$. Aggregation for integration score gives a higher weight to cell-type silhouette score, emphasizing the higher importance of preserving biological information over batch mixing. Higher weight to macro *F*1 in the annotation score reflects a balanced annotation accuracy for both major and non-major cell types.

### Reporting summary

Further information on research design is available in the Nature Portfolio Reporting Summary linked to this article.

## Supplementary information

Hayat_Peer Review File

Supplementary Information

Description of Additional Supplementary Files

Supplementary Data 1

Supplementary Data 2

Supplementary Data 3

Supplementary Data 4

Supplementary Data 5

Supplementary Data 6

Reporting Summary

## Data Availability

All datasets used in this study are publicly available (Supplementary Data 5). The use of these datasets, either as reference or query data, is also specified in Supplementary Data 5. Raw data can be found through their associated publications. Ready-to-use data are available for some datasets, and downloadable links are provided in Supplementary Data 5. Source data underlying the main figures are presented in Supplementary Data 6.

## Code availability

The source code of MASI including analyses of key results in the study can be found at https://github.com/hayatlab/MASI^80^. Processed data required for reproducing major results can also be found at MASI GitHub. Source data for making major figures in this study is available in Supplementary Data 6. To reproduce each major figure in this study, please follow tutorials deposited at https://github.com/hayatlab/MASI/tree/main/tutorial.
