# Supplementary material for: MASI enables fast model-free standardization and integration of single-cell transcriptomics data
Source: Commun Biol. 2023 Apr 28;6:465. doi: 10.1038/s42003-023-04820-3 (PMC10144903; doi:10.1038/s42003-023-04820-3)
Supplement: Supplementary file 3 — Description of Additional Supplementary Files [file 42003_2023_4820_MOESM3_ESM.pdf]

Description of Additional Supplementary Files

File Name: Supplementary Data 1
Description: Integration ranking of 16 data processing pipelines.

File Name: Supplementary Data 2
Description: Evaluation of human heart atlas annotation. Human heart atlas data was annotated at both high and low hierarchy. Macro F1 scores and overall accuracies were reported for every method, except Seurat.

File Name: Supplementary Data 3
Description: Performance ranking of 7 integration methods. 3 different rankings were given, integration, annotation, and overall performance, respectively.

File Name: Supplementary Data 4
Description: Comparison of MASI running with different memory limits.

File Name: Supplementary Data 5
Description: Description of data used. Brief description, including species, tissues, usage of data in this study, GEO accessions, etc. are listed

File Name: Supplementary Data 6
Description: Source data underlying main figures.
