# Supplementary material for: MASI enables fast model-free standardization and integration of single-cell transcriptomics data
Source: Commun Biol. 2023 Apr 28;6:465. doi: 10.1038/s42003-023-04820-3 (PMC10144903; doi:10.1038/s42003-023-04820-3)
Supplement: Supplementary file 1 — Hayat_Peer Review File [file 42003_2023_4820_MOESM1_ESM.pdf]

nature portfolio

Peer Review File

MASI enables fast model-free standardization and integration of single-cell transcriptomics data

Open Access This file is licensed under a Creative Commons Attribution 4.0 International License, which permits use, sharing, adaptation, distribution and reproduction in any medium or format, as long as you give appropriate credit to the original author(s) and the source, provide a link to the Creative Commons license, and indicate if changes were made. In the cases where the authors are anonymous, such as is the case for the reports of anonymous peer reviewers, author attribution should be to 'Anonymous Referee' followed by a clear attribution to the source work. The images or other third party material in this file are included in the article's Creative Commons license, unless indicated otherwise in a credit line to the material. If material is not included in the article's Creative Commons license and your intended use is not permitted by statutory regulation or exceeds the permitted use, you will need to obtain permission directly from the copyright holder. To view a copy of this license, visit http://creativecommons.org/licenses/by/4.0/.

This manuscript has been previously reviewed at another Nature journal.

Reviewers' comments:

REVIEWER #2:
1. I appreciate that authors have done some significant revision to address my comments, but it seems that it is not completed yet. For example, in the rebuttal letter, there are several places labeled with 'TODO' or 'Figure xx', or just an empty ''.

2. Choosing the pipeline #14 is still questionable as it is not so consistent across the datasets tested, e.g., the mouse liver and human pancreas datasets. The authors should use the well accepted batch mixing algorithm, i.e., kBET (https://www.nature.com/articles/s41592-018-0254-1) instead of using batch mixing entropy score to evaluate mixability.

3. Not preforming scRNA-seq preprocessing on the raw data maybe a problem as there are many different scRNA-seq platforms/protocols, each needs to be pre-processed depending on the protocol/platform/technology, before performing normalization and batch correction.

4. The quality of figures is low, some with corrections/marks.

REVIEWER #3:
Although the authors have clarified their presentation, I am still not convinced that the proposed work constitutes a meaningful advance in the field. The authors acknowledge that their method does not perform much better than existing methods, but that this comes with the benefit of using a more simple, transparent approach which also allows for lower computational costs. The authors argue that existing methods "require either large memory ro a high-end GPU". However, the only evidence provided in support of this claim is figure S14 which shows only the runtime and not the memory usage. Moreover, the benchmarking is still limited in my opinion as there is a large number of methods available for this task (see Abdelaal et al, Genome Biology for an old benchmark). In particular, if the selling point of your method is that it is fast, then you should make sure to include other methods in your benchmark that are also optimized for this purpose.

Reviewers' comments:

Reviewer #2 (Remarks to the Author):

1. I appreciate that authors have done some significant revision to address my comments, but it seems that it is not completed yet. For example, in the rebuttal letter, there are several places labeled with 'TODO' or 'Figure xx', or just an empty ''.

These unfinished signs were left in our previous response letter by mistake. We have removed them.

2. Choosing the pipeline #14 is still questionable as it is not so consistent across the datasets tested, e.g., the mouse liver and human pancreas datasets. The authors should use the well accepted batch mixing algorithm, i.e., kBET (https://www.nature.com/articles/s41592-018-0254-1) instead of using batch mixing entropy score to evaluate mixability.

In our manuscript, we have written down our reasoning of selecting pipeline #14. The original text in manuscript is shown below:

"These results also suggest that conversion from gene expression matrix to cell-type score matrix is the core component for integration analysis in our previous study. Pipelines #7 to #14 showed substantial improvement from pipeline #1 to #6, indicating a general usage of cell-type score for single-cell data integration (Supplementary Fig. 1b). In summary, one can convert gene expression matrix to cell-type score matrix with CellMarker (pipeline #11), PanglaoDB (pipeline #12), ScType (pipeline #13), or a specific reference data (pipeline #14) for batch-effect correction and data annotation. Besides these 3 marker databases (CellMarker, PanglaoDB, and ScType), there are also other resources that provide comprehensive or customized cell-type marker information. For instance, cell-type markers identified from bulk RNA-seq database like ImmGenData and HumanPrimaryCellAtlasData. Though the ability to remove batch effects with the other resources is not tested here, we still encourage users to select more customized cell-type marker databases based on their single-cell data source and specific needs. **Since our focus in this study is annotating and integrating scRNA-seq data with reference data, we chose data processing pipeline as #14 as the final data processing pipeline because of its good balance of cell-type silhouette and batch entropy mixing scores**."

Our method takes raw count matrices from different studies as the input. Briefly, Supplementary Figure 1 shows how we performed data processing and transformations (e.g., log normalization, TF-IDF transformation). In all our benchmarks to choose the most optimal pipeline, and then for generating results, we applied the pipelines consistently across datasets and case studies. We cannot agree with the reviewer that pipeline #14 is not so consistent across the datasets tested. Regarding the ability to mix batches and preserve cell-type structure, pipeline #14 achieved good balance across all 6 benchmark datasets (Supplementary Fig. 1b).

Furthermore, Batch mixing entropy score is a well-accepted evaluation metric for batch mixing too. Batching mixing entropy score was first proposed by Haghverdi et al. *Nature Biotechnology* 2018 (https://www.nature.com/articles/nbt.4091), **which estimates batch mixing at k-nearest**

neighbor's scale. Batch mixing entropy score zooms in to a k-nearest neighbor's scale (k=100) and calculates proportion of each batch in this scale. Calculation of entropy with the proportion gives a perspective of how well batches are mixed. Higher entropy would mean better mixing. kBET is similar, in terms of that it also gives estimation for batch mixing at k-nearest neighbor's scale. Batch mixing entropy score, then, has been used by other studies to benchmark single-cell data integration, including Xiong et al. *Nature Communications* 2022 (https://www.nature.com/articles/s41467-022-33758-z) and Cao et al. *Nature Communications* 2022 (https://www.nature.com/articles/s41467-022-35094-8). Meanwhile, we did not only evaluate data integration with batch mixing entropy score in our study, but also used macro F1 score. We believe that good integration should also empower accurate label transferring from reference data to query data. Thus, our conclusion is not solely on how well batches are mixed, but also how accurate the label transferring could be.

3. Not preforming scRNA-seq preprocessing on the raw data maybe a problem as there are many different scRNA-seq platforms/protocols, each needs to be pre-processed depending on the protocol/platform/technology, before performing normalization and batch correction.

We followed common steps like log-transformation, normalization and selecting highly-variable genes as preprocessing steps for scRNA-seq data. For each method in benchmark study, we also did data processing each method suggested by those methods before we ran that method for data integration (see "Data preprocessing" and other method-specific paragraphs in Method section).

4. The quality of figures is low, some with corrections/marks.

We checked every figure in our manuscript and made sure they have good image quality. Additionally, we are able to provide even higher resolution images to the journal if needed. We did not make any corrections to the figures. In Figure 4f, we show a zoomed in figure to focus on a specific cell-type in the overall UMAP.

Reviewer #3 (Remarks to the Author):

Although the authors have clarified their presentation, I am still not convinced that the proposed work constitutes a meaningful advance in the field. The authors acknowledge that their method does not perform much better than existing methods, but that this comes with the benefit of using a more simple, transparent approach which also allows for lower computational costs. The authors argue that existing methods "require either large memory or a high-end GPU". However, the only evidence provided in support of this claim is figure S14 which shows only the runtime and not the memory usage. Moreover, benchmarking is still limited in my opinion as there is a large number of methods available for this task (see Abdelaal et al, Genome Biology for an old benchmark). In particular, if the selling point of your method is that it is fast, then you should make sure to include other methods in your benchmark that are also optimized for this purpose.

In combination with optimal performance on 4 criteria (F1 score, overall accuracy, sillhouhette score, and batch mixing entropy score), on 6 different datasets, we show that our method is still able to run on a) limited computational resources (meaning no GPUs required) and b) is transparent in its approach toward which marker genes are involved in the integration and cell-type annotation. The reviewer may have pointed to a wrong benchmark study in *Genome Biology*. Benchmark study from Abdelaal et al. *Genome Biology* 2019 concluded that SVM has the best performance of cell type classification across different scRNA-seq data. In the task of cell type annotation, we indeed included SVM classifiers for benchmark and showed that MASI has better performance than SVM. Benchmark study from Tran et al. *Genome Biology* 2020 concluded that Harmony, LIGER, Seurat and Scanorama are top methods for batch correction. We also included these 4 methods for task of data integration. Instead of using Harmony, we used its updated version, Symphony.

Most standard laptops do not come equipped with a high-end GPU. However, to be fair to all methods that we benchmarked, we ran all methods on the same device that is equipped with 10 cores Intel Xeon Silver 4210, 64GB memory, and an Nvidia RTX Quadro 6000 (a high-end GPU). Given the same computational resource, different integration methods have their own approach to solve the problem of data integration. The runtime of all these methods is shown in Supplementary Figure 14. Both LIGER and Seurat heavily rely on memory to do large scale matrix manipulation. Thus, these two methods have very high memory usage and cannot be used for large datasets in our hands, due to limitation of RAM memory. Both scNym and scArches are neural network-based methods. Such methods take advantage of the GPU for model training. As training is done by splitting data into mini batches, the memory usage won't explode to the device limit (64GB). We did not try to run scNym and scArches on a CPU-only laptop. Thus, when a GPU is available, scNym and scArches can overcome this limitation by taking advantage of GPUs. Additionally, we measured average memory usage over runtime (See table below). Symphony, Scanorama, LIGER, Seurat, and MASI use a larger proportion of memory in order to accommodate large-scale data integration. As we mentioned earlier, both scArches scANVI and scNym are neural-network-based methods and take advantage of the GPU, and thus both these methods use relatively less computational RAM memory. Considering the huge price difference between RAM memory and GPU, we think making full use of RAM memory is a more affordable solution than making full use of GPU.

Average memory usage over runtime (%)									
# of cells	9714	14801	31774	113018	251057	398523	486134	978734	1196523
MASI	12	12	12	36	43	76	87	92	92
scNym	8	8	8	14	14	37	48	60	60
scArches scANVI	8	8	8	15	19	41	56	64	64
Symphony	10	10	12	24	29	36	64	84	84
Scanorama	8	8	8	16	45	80	88	96	96
LIGER	8	8	8	37	86	96	96	NA	NA
Seurat	8	8	16	35	86	NA	NA	NA	NA
Device equipped with 64GB memory									

Regarding MASI, we refactored it to make full use of the available CPUs (Figure 2c). MASI does not use GPU on this device at all, while it can still perform large scale scRNA-seq data integration as GPU-based methods such as scNym and scArches do. MASI overcomes memory limitation by reducing the batch size. In Supplementary Table 2, we show that when the computational

device only has 16GB memory, we can reduce batch size to overcome memory limitation and make MASI run without sacrificing performance. In summary, using a GPU equipped laptop, we show that some methods need a GPU to be able to integrate large datasets, while others can be run using just the standard CPU. As shown here, MASI gives comparable or better performance to other methods, including methods that rely on GPUs. We aim to highlight that MASI can be run to integrate large single-cell datasets in a transparent manner without large memory requirements and GPU.

Reviewers' comments:

Reviewer #3 (Remarks to the Author):

The authors have addressed my remaining comment and I look forward to seeing the manuscript in print.

Reviewer #4 (Remarks to the Author):

For Reviewer #2's 2nd comment: I think it's qualitative to say pipeline #14 has a "good balance of cell-type silhouette and batch entropy mixing scores". The authors may calculate the average of the aforementioned two metrics or a justifiable weighted average across the six benchmarking datasets. #14 can be selected as long as it's significantly better than the other pipelines.

Similarly, for Reviewer #3's comment, the authors need to show that MASI is significantly better than other existing methods. I do not think limiting memory to 64GB makes much sense. While it's nice to compute on a laptop, nowadays high-performance computing servers are readily available with hundreds of GB of memory.

Reviewer #4 (Remarks to the Author):

For Reviewer #2's 2nd comment: I think it's qualitative to say pipeline #14 has a "good balance of cell-type silhouette and batch entropy mixing scores". The authors may calculate the average of the aforementioned two metrics or a justifiable weighted average across the six benchmarking datasets. #14 can be selected as long as it's significantly better than the other pipelines.

To report aggregated scores and rank all pipelines across benchmark datasets, we selected a similar weighted averaging as used by another single-cell benchmark study by Luecken et al., Nature Methods, 2022 (https://www.nature.com/articles/s41592-021-01336-8). The overall integration score is defined as $S_{integration} = 0.7 \times S_{cell-type} + 0.3 \times S_{batch-mixing}$. This aggregation gives a higher weight to $S_{cell-type}$, emphasizing higher importance of preserving biological information. Then, we ranked all 16 pipelines based on $S_{integration}$ across 6 benchmark datasets (**Supplementary Table 1**). Pipeline #14, indeed, presents better performance than others on the benchmark datasets.

Similarly, for Reviewer #3's comment, the authors need to show that MASI is significantly better than other existing methods. I do not think limiting memory to 64GB makes much sense. While it's nice to compute on a laptop, nowadays high-performance computing servers are readily available with hundreds of GB of memory.

We added a summarized performance table (**Supplementary Table 3**) to show that MASI is consistently better than other existing methods. We calculated a weighted score to give a more concise performance report. This way of weighted averaging is based on Luecken et al., *Nature Methods*, 2022. For the task of integration, an integration score is defined as $S_{integration} = 0.7 \times S_{cell-type} + 0.3 \times S_{batch-mixing}$. For the task of annotation, an annotation score is defined as $S_{annotation} = 0.7 \times F1_{macro} + 0.3 \times ACC_{overall}$. Integration score gives a higher weight to cell-type silhouette score, emphasizing higher importance of preserving biological information over batch mixing. Higher weight to macro F1 in annotation score highlights a balanced annotation accuracy for both major and non-major cell types. Finally, an overall performance score is calculated based on $S_{overall} = 0.5 \times S_{integration} + 0.5 \times S_{annotation}$. In this summarized performance report, MASI was ranked one of top methods across 6 benchmark datasets, in both tasks of data integration and annotation.

Meanwhile, we sincerely appreciate that reviewer brought up the point that nowadays high-performance computing servers are readily available with hundreds of GB of memory. To show that MASI can return good performance outputs when memory is limited, we ran MASI for all 6 benchmark datasets, with 16GB, 32GB, and 64GB maximum memory usage respectively (**Supplementary Table 4**). As data size increases, we need to reduce the batch size from 50000 cells per batch for 64GB to 20000 cells per batch for 16GB, in order to run MASI without exceeding memory limit. We showed that given limited computational resources, specifically low RAM memory, MASI can still perform performance integration and annotation tasks without sacrificing accuracy (**Supplementary Table 4**).

Meanwhile, one of our authors was a PhD student based in a US university. From his personal experience, accessing a good computational resource is not simple as asking for a small favor.

When a PhD student initializes their own research and needs resources to conduct their project, there could be many different reasons that prevent them from accessing these resources. It might be due to resource distribution priority within the institute, a limited research budget, school policy, etc. In this study, a workstation equipped with a decent graphic card and a 64GB memory is a real gift for us to perform benchmark and method testing in this study. If researchers in well-developed countries like US still face difficulties of accessing computational resources, not mention those in developing countries and in their early career stage may even face harder situation, in order to allocate their research resource wisely.
